# The hypoxia-sensor carbonic anhydrase IX affects macrophage metabolism, but is not a suitable biomarker for human cardiovascular disease

**DOI:** 10.1038/s41598-020-79978-5

**Published:** 2021-01-11

**Authors:** J. A. F. Demandt, L. J. Dubois, K. van Kuijk, M. Zaťovičová, H. Jin, S. Parkkila, S. W. van der Laan, L. Jelenska, B. M. E. Mees, C. P. M. Reutelingsperger, K. B. J. M. Cleutjens, C. J. H. van der Kallen, C. G. Schalkwijk, M. M. J. van Greevenbroek, E. A. L. Biessen, G. Pasterkamp, S. Pastoreková, C. D. A. Stehouwer, J. C. Sluimer

**Affiliations:** 1 Cardiovascular Research Institute Maastricht (CARIM), Maastricht University Medical Center (MUMC), Maastricht, The Netherlands; 2 Department of Pathology, MUMC, P. Debyelaan 25 6229HX Maastricht, The Netherlands; 3 Department of Precision Medicine, The M-Lab, GROW Research Institute for Oncology, MUMC, Maastricht, The Netherlands; 4 Biomedical Research Center of the Slovak Academy of Sciences, Bratislava, Slovak Republic; 5 Faculty of Medicine and Health Technology, Tampere University, Tampere, Finland; 6 Fimlab Ltd, Tampere University Hospital, Tampere, Finland; 7 Division Laboratories, Pharmacy, and Biomedical Genetics, Central Diagnostics Laboratory, University Medical Center Utrecht, Utrecht University, Utrecht, The Netherlands; 8 Department of Vascular Surgery, MUMC, Maastricht, The Netherlands; 9 Department of Biochemistry, MUMC, Maastricht, The Netherlands; 10 Department of Internal Medicine, MUMC, Maastricht, The Netherlands; 11 Institute for Molecular Cardiovascular Research, RWTH Aachen University, Aachen, Germany; 12 BHF Centre for Cardiovascular Sciences (CVS), University of Edinburgh, Edinburgh, UK

**Keywords:** Atherosclerosis, Cell death and immune response, Prognostic markers

## Abstract

Hypoxia is prevalent in atherosclerotic plaques, promoting plaque aggravation and subsequent cardiovascular disease (CVD). Transmembrane protein carbonic anhydrase IX (CAIX) is hypoxia-induced and can be shed into the circulation as soluble CAIX (sCAIX). As plaque macrophages are hypoxic, we hypothesized a role for CAIX in macrophage function, and as biomarker of hypoxic plaque burden and CVD. As tumor patients with probable CVD are treated with CAIX inhibitors, this study will shed light on their safety profile. CAIX co-localized with macrophages (CD68) and hypoxia (pimonidazole), and correlated with lipid core size and pro-inflammatory iNOS+ macrophages in unstable human carotid artery plaques. Although elevated pH and reduced lactate levels in culture medium of CAIX knock-out (CAIXko) macrophages confirmed its role as pH-regulator, only spare respiratory capacity of CAIXko macrophages was reduced. Proliferation, apoptosis, lipid uptake and expression of pro- and anti-inflammatory genes were not altered. Plasma sCAIX levels and plaque-resident CAIX were below the detection threshold in 50 and 90% of asymptomatic and symptomatic cases, respectively, while detectable levels did not associate with primary or secondary events, or intraplaque hemorrhage. Initial findings show that CAIX deficiency interferes with macrophage metabolism. Despite a correlation with inflammatory macrophages, plaque-resident and sCAIX expression levels are too low to serve as biomarkers of future CVD.

## Introduction

Atherosclerotic plaque development and subsequent rupture are pivotal in the development of clinical cardiovascular disease (CVD), and orchestrated by close interplay between inflammation and cholesterol. Abundant plaque macrophages require vast amounts of oxygen, which is scantily present in the plaque core^1–3^. Macrophage hypoxia is thought to make plaques prone to intraplaque hemorrhage (IPH) and rupture, subsequently leading to clinical thrombotic events. Plaque hypoxia imaging with [^18^F]-HX4 could identify human plaques with traits associated to rupture^2^, hence plasma biomarkers of plaque hypoxia may offer a cost-effective alternative to identify patients with rupture-prone atherosclerotic plaques.

To this end, carbonic anhydrase IX (CAIX) could be a suitable biomarker, as it is a hypoxia-induced transmembrane protein of which the extracellular 4 kDa component can be shed into body fluids by a disintegrin and metalloprotease (ADAM)17^4^. Under physiological conditions, it is mainly expressed in stomach and proximal intestinal epithelial cells^5,6^. CAIX function and the biomarker potential of soluble CAIX (sCAIX) to asses tumor hypoxia have been widely studied in the context of cancer^7–10^. In short, cancer cells adapt to glycolysis as their main source of ATP, regardless of oxygen availability, known as the Warburg effect^11^. This results in pericellular acidification, promoting cancer cell proliferation, migration and invasion^12,13^. Moreover, the metabolically adapted cancer cells become resistant to radiation and chemotherapy induced cell death^14^. CAIX plays an important role in this transition to and maintenance of the Warburg effect and subsequent extracellular acidification, serving as pH sensor and regulator of intra- and extracellular pH^15^. In fact, inhibiting CAIX function was shown to potentiate radiation and chemotherapy in multiple types of cancer, underlining the importance of CAIX in tumor cell survival^9^.

How do these findings of the cancer field translate to macrophage function in the atherosclerotic plaque? First of all, development of plaque hypoxia might induce expression of CAIX and subsequently increase circulating levels of sCAIX. As plaque hypoxia is associated with plaque hemorrhage and instability^1–3^, plaque-resident CAIX or sCAIX could thus be associated with plaque instability. Secondly, the Warburg effect has also been described in activated macrophages^16^, and hypoxic macrophages will thus rely on glycolysis for fuel supply^17^. Furthermore, in advanced human carotid plaques, pH was shown to be as low as 6.8^18^, possibly due to CAIX activity, while physiologic interstitial pH is ~ 7.4. Low pH is known to have detrimental effects on atherogenesis. In RAW264.7 macrophages, low pH was sufficient to induce a pro-inflammatory macrophage phenotype^19^. In addition, low pH will also increase pericellular matrix degradation, alter lipid homeostasis and hamper macrophage lipid handling^20^. Furthermore, several pro-atherogenic lipid modifications are induced by intraplaque acidification, potentially promoting plaque progression. Indeed, deficiency of another pH regulator, Na+ H+ exchanger 1, led to reduced plaque formation^21^. Altogether this corroborates the hypothesis that plaque acidification, possibly via macrophage CAIX, could promote plaque development and excessive expression of this protein might identify advanced plaques with a hypoxic, and/or acidic milieu. As therapeutics directed against CAIX are pursued in cancer patients^9^, an elderly group with a likely higher burden of CVD, the role of CAIX in atherosclerosis should be investigated to assess the CVD safety profile. In addition, CAIX and sCAIX could be biomarkers of plaque hypoxia and/or acidity to predict future cardiovascular events. Hence, in this study, we aimed to explore the expression of CAIX in human atherosclerosis, a role for CAIX in atherogenic macrophage functions and its potential as a biomarker for atherosclerotic disease.

## Results

### CAIX is present in human atherosclerotic plaques and correlated with pro-atherogenic traits

**Figure 1:**
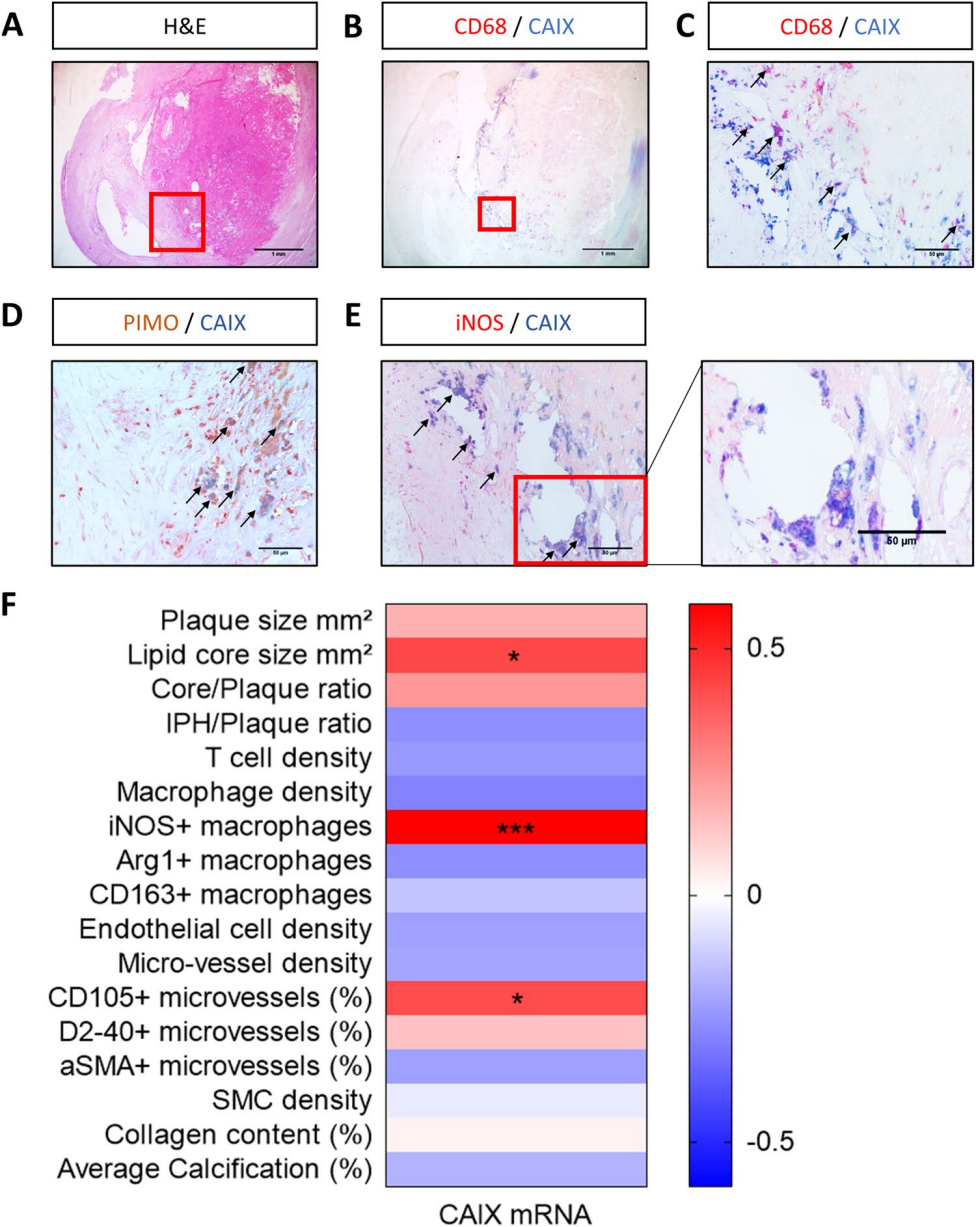
CAIX was present in human atherosclerotic plaque and co-localized with CD68^+^ cells. (**A**) Microphotograph of hematoxylin and eosin (H&E) stained human unstable carotid artery plaque. Red square represents region that was magnified in all stainings. (**B**) Double staining of CAIX (blue) and CD68 (red) in unstable human atherosclerotic plaque, magnification of red square is depicted in (**C**). Arrows indicate double positive cells. (**D**) Unstable human atherosclerotic plaque of patient injected with pimonidazole to detect hypoxia (brown) or (**E**) iNOS (red), and CAIX (blue), arrows indicate double-positive areas accompanied by an additional zoomed in area, (**F**) Heatmap of Pearson correlations of CAIX mRNA in unstable human plaque segments with plaque traits, determined on adjacent histology slides. All plaque sections originate from the MaasHPS cohort. Arg1; arginase 1. αSMA; alpha smooth muscle cell actin. D2-40; podoplanin. iNOS; inducible nitric oxide synthase. IPH; intra-plaque hemorrhage. MVD, microvessel density NA; not applicable. *p < 0.05, **p < 0.01, ***p < 0.001.

As the expression pattern of CAIX in atherosclerosis was unknown, and macrophages are the predominant hypoxic cells expected to express CAIX, we performed double immunohistochemical staining for CAIX and CD68 to evaluate their co-localization in human unstable carotid plaques (Fig. 1A–C). CAIX immunoreactivity was detected on only 5–10% of the plaque surface area (Fig. 1B, Supplemental Figure S2). If present, CAIX was observed in the macrophage-rich shoulder regions of the plaque, as well as in the thick fibrous cap. CD68-negative, spindle-like cells reflecting smooth muscle cells and/or fibroblasts were also positive for CAIX. As expected, CAIX expression was positive in pimonidazole-positive, hypoxic regions in the human plaque (Fig. 1D). Furthermore, *CAIX* mRNA expression in unstable human plaques correlated with pro-inflammatory macrophages (iNOS/CD68), lipid core size, and CD105+ new angiogenic microvasculature (Fig. 1F). The former was validated by a double staining for iNOS and CAIX, showing co-expression by foamy cells in the hypoxic plaque region as indicated by the arrows (Fig. 1E). The correlation with new angiogenic sprouts, likely also a response to hypoxia, might reflect the hypoxia-responsive nature of CAIX. These data suggested an association of CAIX with plaque hypoxia and inflammation, and warranted further investigation of a role in macrophage function, and potential as biomarker.

### CAIX deficiency alters macrophage metabolism

From the extensive literature available from the cancer field, we know that CAIX is involved in pH regulation, thereby enabling metabolic changes in cancer cells. As we observed a correlation with iNOS+ macrophages, we reasoned that CAIX deficiency might also interfere with macrophage metabolism and polarization, in this way affecting atherosclerosis relevant functions. Therefore we isolated BMDM from CAIXko mice^22^. First, we confirmed gene knock-out in stomach tissue from CAIXko mice compared to WT mice (Supplemental Figure S1).

**Figure 2:**
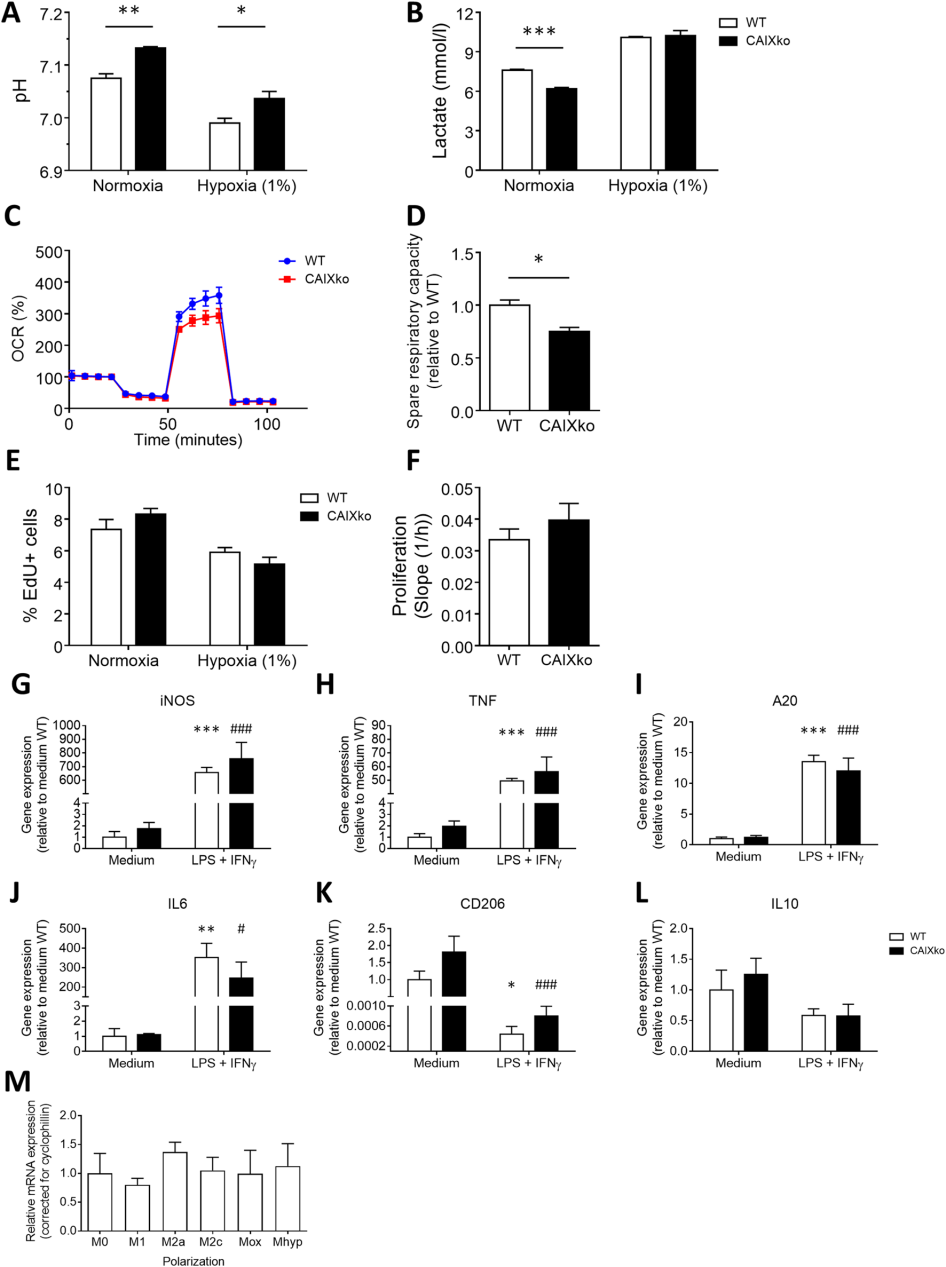
CAIXko reduced spare respiratory capacity in BMDMs. (**A**) pH and (**B**) lactate measurement of BMDM culture medium (n = 4) after 72 h of incubation in either normoxia or hypoxia (1% O_2_). (**C**) Representative graph of Seahorse mitochondrial stress assay of BMDMs (n = 5/group, 1 experiments). Analysis was performed on data corrected for protein content in each well and with baseline measurement 4. (**D**) BMDM spare respiratory capacity displayed relatively to WT. (**E**) BMDM proliferation measured by EdU incorporation (n = 4) after 24 h incubation in normoxia or hypoxia (1% O_2_). (**F**) Mean BMDM proliferation measured by real-time impedance (n = 5) over 72 h. Slope represents the increment of impedance over time. (**G**) Real time quantitative PCR of pro-inflammatory genes inducible nitric oxide synthase (iNOS), (**H**) tumor necrosis factor (TNF), (**I**) Tumor necrosis factor-induced protein 3 (A20) (**J**) interleukin 6 (IL6) and anti-inflammatory genes (**K**) mannose receptor (CD206) and (**L**) interleukin 10 (IL10) in non-stimulated (medium) and LPS + IFNγ stimulated BMDMs. All values are relative to WT unstimulated BMDMs of respective target gene. *Indicates a significant difference of stimulated WT compared to unstimulated WT BMDM. #Indicates significant difference of stimulated CAIXko compared to unstimulated CAIXko BMDMs. No difference was observed between WT and CIX-ko BMDM (white and black bars respectively). All results show mean ± SEM. *p < 0.05, **p < 0.01, ***p < 0.001.

Thereafter, we assessed if CAIX also harbors a function in pH regulation in BMDMs. As expected, the pH of CAIXko BMDM cell culture supernatant was enhanced, and lactate content was lowered, compared to control BMDMs, confirming the functional knockout of CAIX in BMDM (Fig. 2A,B). Energy metabolism was assessed using the seahorse XF analyzer. CAIXko BMDMs were not able to produce similar oxygen consumption rates as WT BMDM upon mitochondrial oxidative phosphorylation uncoupling. This so-called respiratory spare capacity was significantly lowered in CAIXko BMDMs, while baseline ATP production was not altered (Fig. 2C,D). Nevertheless, the reduction of respiratory capacity did not affect cell proliferation measured by impedance and EdU incorporation (Fig. 2E,F).

Macrophages undergo several metabolic changes in order to polarize successfully towards pro- or anti-inflammatory phenotypes^23,24^ and *CAIX* mRNA expression also correlated with pro-inflammatory macrophage presence in human plaque (Fig. 1F). We thus assessed markers of pro- and anti-inflammatory macrophages on mRNA level in BMDMs treated with LPS + IFNγ. No significant change in pro- and anti-inflammatory gene expression was observed between CAIXko and WT cells (Fig. 2G–L), nor did M1 or M2 cytokines change CAIX expression (Fig. 2M). Together, these data support a role for CAIX in pH regulation and a lower ability of their oxidative phosphorylation machinery to respond to a sudden increase in energy demand.

### CAIXko macrophages do not show a distinct pro-atherogenic phenotype

**Figure 3:**
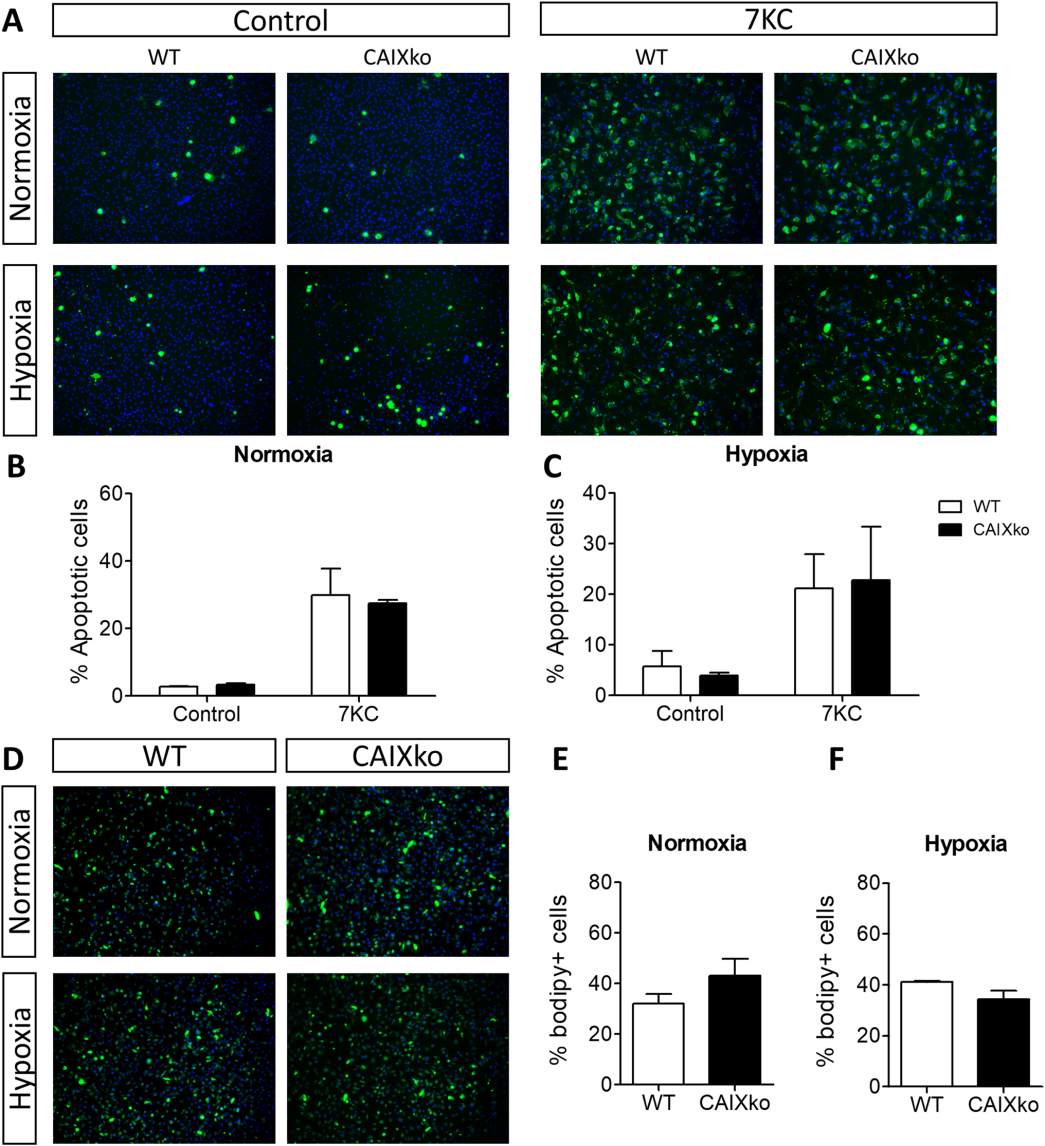
CAIXko did not alter BMDM apoptosis or lipid uptake. Apoptosis was induced by incubating cells with 50 µM 7-ketocholesterol for 24 h in all experiments. (**A**) Representative photographs of apoptotic cells (annexin positive) in WT and CAIXko BMDMs in normoxia and hypoxia (1% O_2_). (**B**) BMDM (n = 3) apoptosis in normoxia or (**C**) hypoxia (1% O_2_, 24 h). (**D**) Representative photographs of BMDMs after lipid uptake (Topfluor positive) in WT and CAIXko BMDMs in normoxia and hypoxia (1% O_2_). (**E**) BMDM lipid uptake in normoxia (n = 3) or (**F**) hypoxia. All results show mean ± SEM.

Since *CAIX* mRNA correlated with pro-atherogenic plaque traits, and CAIXko led to metabolic changes in BMDMs, we further investigated its role in macrophage functions relevant for atherosclerosis. As expression of *CAIX* mRNA in human unstable plaque segments correlated with lipid-rich necrotic core size, we explored BMDM apoptosis and lipid uptake. BMDM apoptosis in response to 7-ketocholesterol, was unaffected in both normoxia and hypoxia (Fig. 3A–C). As confirmation, there was no clear correlation between *CAIX* mRNA and genes involved in apoptosis in human unstable plaque segments. Only 10 of 158 genes included in the “hallmarks of apoptosis” geneset derived from gene set enrichment analysis were significantly correlated with CAIX mRNA (Supplemental table S1). In addition, lipid uptake by CAIXko BMDMs was not significantly altered both in normoxia and hypoxia (Fig. 3D–F). Together, CAIXko in macrophages did not change the atherosclerosis-relevant functions studied here, despite having small changes in metabolism.

### Baseline sCAIX did not associate with future primary cardiovascular disease outcome measures

Although a role for CAIX in macrophage function seemed limited, its expression patterns and the known hypoxia-selective shedding, suggested to explore whether the soluble CAIX (sCAIX) in plasma or plaque-resident CAIX were associated with cardiovascular outcome measures. We studied the association between plasma sCAIX and CVD in 579 asymptomatic participants with future, primary events in the Cohort On Diabetes and Atherosclerosis Maastricht (CODAM).

First, plasma of CODAM was collected at inclusion of asymptomatic patients, and plasma CAIX measured at baseline was associated with future, primary events during 7 years follow-up. Plasma sCAIX was detectable in only 14% of participants from the CODAM cohort, and there was no significant association between the detectability of sCAIX, and cardiovascular outcome measures (Supplemental table S2). As can be appreciated from Table 1 and Supplemental table S3, CAIX was unable to predict prevalence or incidence of CVD or CVE in the CODAM, regardless of the model. We then investigated a potential relationship between sCAIX and plaque burden, as it is well conceivable that enhanced total body plaque burden would lead to enhanced circulating levels of sCAIX. We thus used intima-media thickness of the carotid artery (cIMT) and ankle-arm index (AAIx) as measures for carotid and peripheral artery plaque burden, respectively. However, linear regression analysis did not reveal an association between sCAIX and plaque burden (Table 2).

**Table 1:** Association of sCAIX with prevalent CVD and CVE.

CVD	OR	95%; CI	*p*-value
Model 1	0.80	0.461; 1.401	0.441
Model 2	0.72	0.405; 1.277	0.261
Model 3	0.97	0.510; 1.837	0.921

CVE	OR	95%; CI	*p*-value
Model 1	0.75	0.370; 2.525	0.428
Model 2	0.66	0.317; 1.281	0.271
Model 3	1.07	0.430; 2.671	0.881

Logistic regression analysis using 572 participants for model 1 and 2, 560 participants for model 3, s packyears had 12 missing values. sCAIX was treated as dichotomous independent variable (detectable yes/no, yes, n = 80). Model 1: Crude, no adjustments. Model 2: model 1 + adjustment for sex and age. Model 3: model 2 + adjustments for smoking (status [current, former, never] & packyears), medication (lipid-modifying y/n, anti-HT y/n, glucose-lowering y/n), glucose metabolism status (IGM y/n, DM2 y/n).

**Table 2:** Association of sCAIX with cIMT and AAIx.

cIMT	β	95%; CI	*p*-value
Model 1	0.027	− 0.011; 0.066	0.166
Model 2	0.021	− 0.016; 0.058	0.275
Model 3	0.026	− 0.012; 0.064	0.185

AAIx	β	95%; CI	*p*-value
Model 1	− 0.006	− 0.037; 0.024	0.689
Model 2	− 0.001	− 0.031; 0.028	0.938
Model 3	− 0.009	− 0.038; 0.020	0.550

Linear regression analysis using 504 and 541 participants that underwent cIMT and AAIx measurement, respectively. sCAIX was treated as dichotomous independent variable (detectable yes/no). β indicates mutation of dependent variable if CAIX is detectable (yes). Model 1: Crude, no adjustments. Model 2: model 1 + adjustment for sex and age. Model 3: model 2 + adjustments for smoking (status [current, former, never] & packyears), medication (lipid-modifying y/n, anti-HT y/n, glucose-lowering y/n), glucose metabolism status (IGM y/n, DM2 y/n).

As hypoxic tumors are a known source of sCAIX^25–27^, we assessed cancer prevalence. The presence of cancer could not have influenced our data, since 17 (3.5%) participants with undetectable sCAIX reported active cancer, compared to 3 participants with detectable levels (3.8%). Together, sCAIX levels were frequently undetectable excluding it as a meaningful biomarker of cardiovascular disease outcome measures in the CODAM cohort, and this was not biased by the presence of hypoxic tumors.

### sCAIX levels were similar in symptomatic patients with and without intraplaque hemorrhage

**Figure 4:**
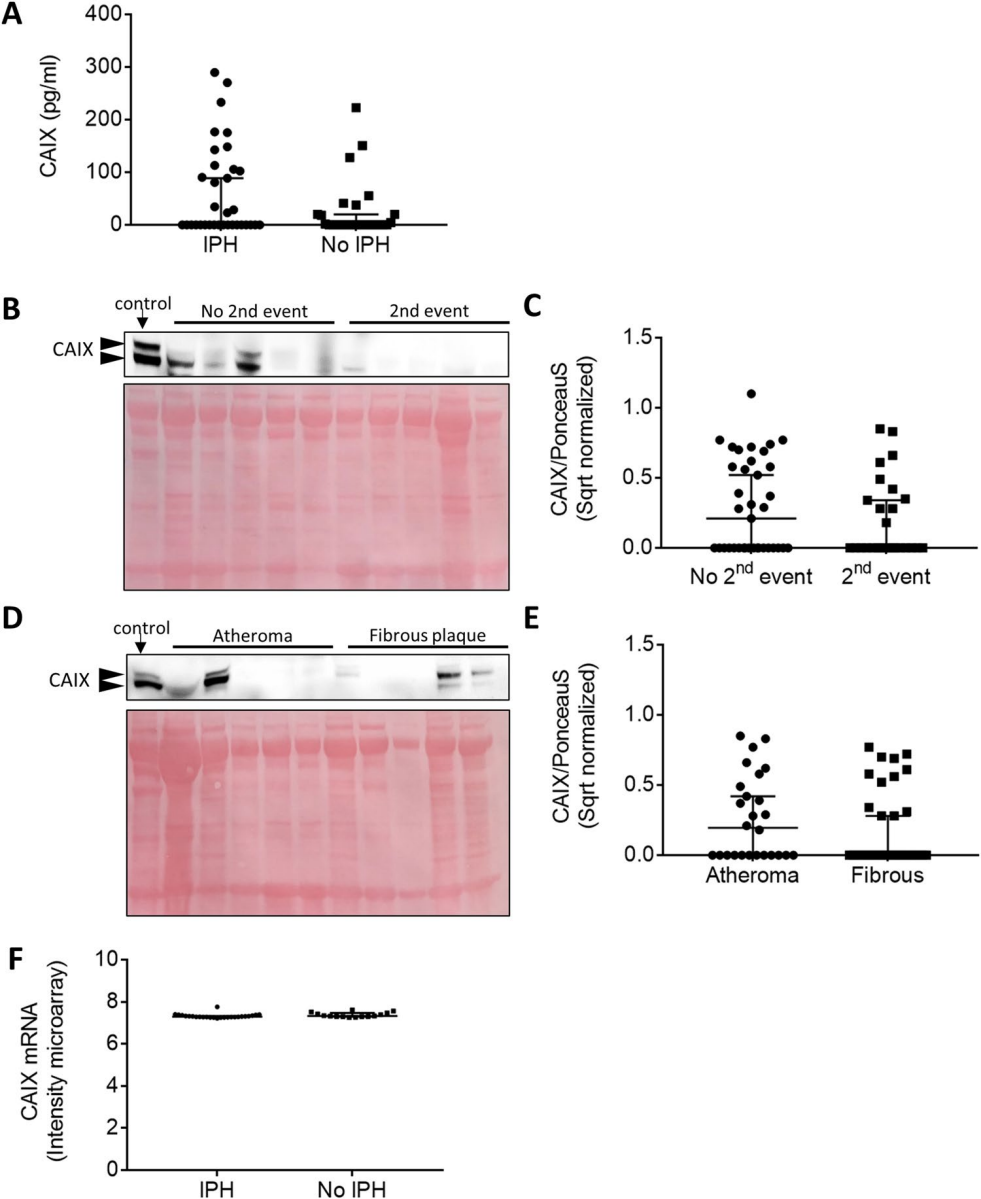
sCAIX and plaque resident CAIX was not changed in patients with events or plaque type. (**A**) sCAIX values in human carotid plaques with or without IPH from the MaasHPS cohort. (**B**) Representative cropped western blot and **(C)** quantification of CAIX protein in human carotid plaque lysates with (n = 27) and without a second event (n = 35) derived from Athero-Express biobank study. CAIX protein intensity is corrected for protein levels by ponceau S, and square root (sqrt) normalized. (**D**) Representative cropped western blot and (**E**) quantification of CAIX protein in atheromatous (26) and fibrous (n = 33) human carotid plaque lysates derived from Athero-Express biobank study. Arrow heads indicate two bands known to correspond to CAIX, 2nd, second. Full length western blots can be found in Supplemental Figures S3 and S4, (**F**) CAIX mRNA as derived from microarray analysis in human carotid plaques with or without IPH derived from the MaasHPS biobank.

As sCAIX did not predict future, primary CVD or correlated with atherosclerotic burden in patients with asymptomatic disease at the time of plasma collection in the CODAM cohort, we measured sCAIX in plasma samples of recently symptomatic patients with (n = 35) or without histological evidence of intraplaque hemorrhage (IPH) (n = 28) in their carotid plaques, collected during carotid endartectomy (MPTC cohort)^28^. Participants from this cohort were known to have severe, symptomatic atherosclerosis, with expected increase in plaque hypoxia, and hence might be a more suitable population to detect sufficient levels of sCAIX. Therefore, we investigated if sCAIX could distinguish between stages of plaque severity. The detectable fraction of sCAIX was indeed higher in these recently symptomatic patients compared to the participants in CODAM. Unfortunately, sCAIX levels remained largely undetectable in plaques with or without IPH from these symptomatic patients (45.7% and 46.4% respectively), and median levels were also similar (Fig. 4A). In conclusion, there is no difference in sCAIX levels between symptomatic patients presenting with or without IPH in carotid plaques.

### Plaque-resident CAIX protein was similar in participants with and without secondary events

sCAIX could neither predict cardiovascular outcome measures, nor distinguish between patients with stable versus unstable plaques. Interestingly, sCAIX failed to predict disease progression in multiple types of cancer, whereas tumor bound CAIX did^26,27^. Therefore we tested the hypothesis that plaque-resident CAIX does have prognostic value for future CVD, as has been shown for other plaque constituents e.g. osteopontin^29^, and fatty acid-binding protein 4^30^ in the Athero-Express cohort. This cohort consists of plaques collected from symptomatic patients at carotid endarterectomy for plaque phenotyping and protein expression analysis. Participants were followed up to register secondary cardiovascular events, and plaque phenotype and expression of certain proteins were found to predict these secondary events^29–31^. In line with the detectable fraction of sCAIX in symptomatic patients of the Maastricht MPTC plasma cohort, plaque resident CAIX was only detected in 53.1% of plaques. This is in agreement with the non-abundant expression of 5–10% positive surface area of CAIX immunoreactivity in the MaasHPS cohort (Supplemental Figure S2). Moreover, plaque resident CAIX was similar between cases with just a primary event versus cases with a secondary clinical event from any vascular bed, and between unstable plaques with intraplaque hemorrhage (IPH) and stable plaques, or with high or low inflammatory burden (Fig. 4B,C, Supplemental table S4). CAIX protein levels were also similar in lipid-rich, atheromatous plaques with a high inflammatory content compared to lipid-poor, fibrous plaques with high collagen content (Fig. 4D,E). Moreover, CAIX mRNA was also similar between human carotid plaques with and without IPH, albeit in a different patient group (Fig. 4F). Together, expression of plaque resident CAIX was not abundant and if detectable, did not associate with future CVD and did not distinguish between plaque types, in line with sCAIX.

## Discussion

In this study, we used multiple human cohorts and murine macrophages to study expression, function and biomarker potential of CAIX in CVD. CAIX was expressed in human atherosclerotic plaques with intraplaque hemorrhage and co-localized with CD68+, iNOS+ macrophages and hypoxia. In addition, CAIX deficiency in BMDM led to reduced spare respiratory capacity. However, macrophage polarization, lipid uptake properties, or apoptosis rate in normoxia and hypoxia were not affected by CAIX knockout. Given its co-localization with hypoxia, and plaque hypoxia correlating with total lesion burden and lesion vulnerability, we explored the use of plaque-resident CAIX and circulating sCAIX, as a biomarker for cardiovascular disease outcome. However, both CAIX and sCAIX were undetectable in the majority of cases, excluding them as a reliable biomarker. If detectable, CAIX was similar between plaque types, and between participants who were event-free during follow-up versus those with recurrent CVE.

In our study, CAIX mRNA expression correlated with pro-inflammatory iNOS+ macrophages, but not with CD163+ or Arginase + M2 macrophages in atherosclerosis. In contrast, in human cervical cancer CAIX correlated with CD163+ tumor-associated macrophages^32^. A possible explanation may be the known lipid-laden, and pro-inflammatory cell and cytokine environment of the plaque, which greatly facilitates the M1 polarization. Moreover, the plaque contains modified lipids, triggering phagocytic reactions to non-self epitopes. In contrast, mutated, yet endogenous, tumor cells, evade immune cell recognition and phagocytosis by production of suppressive cytokines^33^. A direct comparison of plaque macrophages and tumor-associated macrophages, e.g. by single cell RNA sequencing analysis, could shed more light on their comparison, but is yet lacking.

Despite the association of CAIX with iNOS in human plaques, our study surprisingly fails to show any effect of CAIX deficiency on macrophage polarization. Lipid uptake, proliferation, and apoptosis both in normoxia and hypoxia were also not affected by CAIX deficiency. This also contrasts to tumor CAIX, which correlates with tumor cell survival^34^. Unlike tumor cells, macrophage survival under stress seems independent of CAIX function. Of course, this is not an exhaustive analysis of all macrophage functions pertaining to atherogenesis. Even though we did observe small metabolic effects of CAIXko, this did not interfere with macrophage function in relation to atherogenesis, as evaluated by survival and polarization. It could be the case that discrepancies between in vitro function and in vivo association data are caused by other cells involved in plaque development, as CAIX expression is not limited to macrophages, and mRNA was derived from total plaque lysates. Cellular communication in the atherosclerotic plaque has been shown to influence cell density and reactivity to stimuli^35^. Another explanation could be the inconsistency between CAIX mRNA and protein levels, which has already been indicated in numerous other organs, including kidney, colon and muscle^36^. Although our conclusion on CAIX function is limited to in vitro studies, together with our human in vivo associative results, this suggest only a limited function of CAIX in macrophage biology in the setting of atherosclerosis. In fact, the effect of another pH regulator, Na + H+ exchanger 1, was shown in vitro and in vivo*,* possibly suggesting its dominant role in pH regulation in plaque macrophages^21^.

We investigated the association of sCAIX and plaque-resident CAIX, in view of its tight association with plaque hypoxia, with cardiovascular disease outcome measures as determined in CODAM and in Athero-Express. We did not find any association between sCAIX and pre-specified outcomes in participants of the CODAM cohort. In the majority of cases sCAIX was actually undetectable, which is surprising in light of overt plaque hypoxia expected to upregulate CAIX^1^. As specificity and detection sensitivity of the CAIX ELISA is high (> 30 pg/ml), it is unlikely that the low detection rate is an artifact due to the technology used. In accordance, expression of plaque-resident CAIX was also limited, as demonstrated by immunohistochemistry and western blotting. There seems to be a discrepancy between mRNA and protein levels, which may be partly explained by the high sensitivity of microarray technology to detect mRNA versus lower sensitive protein detection. In addition, CAIX protein stability and/or shedding may be compromised in atherosclerosis. CAIX shedding is regulated by its sheddase ADAM17, whose activity in turn is negatively affected by extracellular acidity and TIMP3 activity^37,38^. ADAM17 is expressed in human atherosclerosis and even higher in unstable plaques^39^. However, its activity may be compromised as plaques indeed show low extracellular pH and TIMP3 expression^40,41^, possibly explaining the high frequency of undetectable sCAIX.

The biomarker potential of sCAIX to predict cardiovascular disease or event risk in a non-acute setting seems limited. Importantly, two recent studies uncovered that circulating sCAIX does not correlate with tissue CAIX expression in non-small lung cancer and oral squamous cell cancer tissue^26,27^. Alternatively, we analysed CAIX in plaque protein lysates of human carotid arteries. The advantage over sCAIX would be that other sources of sCAIX that can ‘pollute’ the measurement, are excluded. Nevertheless, plaque-resident CAIX in carotid plaques obtained after the first CVE were also undetectable in half the cases. Detectable CAIX did not predict recurrence of symptoms over time, nor distinguish between plaque types or plaque traits, like extent of inflammation or IPH in the Athero-Express cohort^42^. CAIX in coronary plaque lysates was not studied, although parameters in the CODAM cohort did reflect overall CVD across vascular beds. Hence, the association of CAIX with hypoxic plaque macrophages might be a mere consequence of plaque hypoxia to prevent intracellular acidification. However, as “*absence of evidence, is not evidence of absence*”, the CAIX and sCAIX levels below the detection threshold of our protein assays do not fully exclude a correlation with human disease progression and clinical events. We can merely conclude that CAIX and sCAIX are not suitable as biomarkers for CVD with currently available assays.

One limitation of the current study is the discrepancies between the cohorts in sCAIX detection. sCAIX was detected in roughly 45% of symptomatic patients (MPTC cohort), while sCAIX was only detected in 14% of asymptomatic patients (CODAM cohort). Discrepancies between the two cohorts reside in the time of plasma collection and symptomatic versus asymptomatic patients. It could very well be that plaque progression, and hence aggravated hypoxia, could influence the presence of sCAIX, however, it is important to note that IPH did not alter sCAIX levels. Although the number of included patients is reasonably higher in the CODAM cohort, it is more likely that clinical parameters, e.g. symptomatic versus asymptomatic, are responsible for the discrepancies between the two cohorts. Another possible limitation of the CODAM cohort for CAIX detection includes the presence of occult malignancies or adiposity among its participants. At moment of inclusion, there was no difference in the presence of self-reported cancer between both groups (i.e. sCAIX detectable, non-detectable). Thus, active cancer probably did not play a role in the detection of sCAIX. Adipose tissue is considered to be hypoxic^43^ due to impaired vascularization, with enhanced levels of HIF1α expression, which could hence be a non-specific source of sCAIX. However, there is also evidence that oxygen tension is not reduced in adipose tissue of obese compared to lean human individuals^44^. Moreover, BMI showed no association with sCAIX in the CODAM study, suggesting limited influence of possible adipose tissue hypoxia on circulating sCAIX. The study is also limited to populations with elderly participants, at high risk of CVE. In addition, the involvement of CAIX in disease initiation was not studied here, while murine plaque macrophages are already hypoxic in early, fatty streaks^3^. Future studies of atherosclerotic plaque initiation in CAIXko mice on a hypercholesterolemic background would yield a definitive answer. Also, investigation of upstream oxygen sensors, like the prolyl hydroxylase enzymes, and their macrophage function, and association with CVD, might provide a better hypoxia biomarker. Alternatively, a combination of hypoxia and/or other markers may be predictive. Together, the conclusions from this study are limited to progression of existing human carotid disease in elderly, high-risk participants. Nevertheless, this is currently most relevant for the clinical practice where established disease is treated. It is also relevant for cancer patients with established CV co-morbidities, being treated with anti-CAIX therapy^9^. The possible lack of association of CAIX with CVD presents a window of CVD safety for this treatment, and relieves concerns of future treatment in the elderly population with multi-morbidities.

In conclusion, CAIXko did not impact BMDM proliferation, apoptosis or lipid uptake, despite small metabolic effects. In addition, circulating sCAIX or plaque-resident CAIX levels were very low and hence not suitable as biomarkers of cardiovascular outcome. CAIX expression is likely a response of hypoxic plaque macrophages without major consequences for future human CVD in elderly, high-risk participants.

## Methods

### Animal tissues

Femur and tibia were obtained from adult CAIXko and WT mice on a C57/Bl6 background from breeding colonies in Oulu University, Finland to culture bone-marrow-derived macrophages. Use of adult CAIXko and WT mice for bone marrow isolation was in concordance with FELASA recommendations and was approved by the Animal Experimentation Committee of the University of Oulu. The CA IX deficient mice have been described and characterized earlier^22,45^. No live animals were used in this study.

### Cell culture

Bone marrow was isolated and cells were cultured for seven days in RPMI-1640 (Gibco with Glutamax, 2 g/L glucose) supplemented with 10% FCS, 100U/ml Penicillin-Streptomycin, and 15% L929-conditioned medium to generate BMDMs. BMDMs were always allowed to attach for 24 h, prior to any additional stimuli. BMDMs were polarized for 24 h to pro-inflammatory M1 macrophages (LPS (10 ng/ml) and IFNγ (100u/ml)), to anti-inflammatory M2A (IL-4, 20 ng/ml) or M2C (IL-10, 10 ng/ml) macrophages or to lipid laden Mox macrophages (oxLDL, 25 µg/ml).

### Apoptosis

BMDMs were stimulated with 50 µM 7-ketocholesterol (Sigma, C2394) for 24 h to induce apoptosis. After stimulation, nuclei were stained with Hoechst (15 µg/ml, Sigma) and apoptotic cells with fluorescently labeled AnxA5-FP488 (produced by Biochemistry department, Maastricht University) for 15 min. Samples were analyzed using a high-throughput, fluorescent reporter system, coupled to automated microscopy (BD Pathway 855 High Content Bioimager). Data was processed with Attovision and BD Diva software.

### Lipid uptake

Isolation of LDL and subsequent oxidation into oxLDL is described elsewhere^46^. Post-dialysis oxLDL concentration was determined using the bicinchoninic acid (BCA) protein assay kit (Pierce, 23227). Cells were either put in hypoxic (1% O_2_) or normoxic culture conditions for 24 h. BMDMs were incubated for 3 h with a mix oxLDL (8 µg/ml) and Topfluor cholesterol (Avanti Polar Lipids, 810255). After wash, nuclei were stained using Hoechst (15 µg/ml, Sigma). Samples were analyzed using a high-throughput, fluorescent reporter system, coupled to automated microscopy (BD Pathway 855 High Content Bioimager). Data was processed with Attovision and BD Diva software.

### EDU incorporation

Cells were exposed to hypoxia (1% O_2_) or normoxia for 24 h. Afterwards, EdU (5-ethynyl-2′-deoxyuridine) (Thermo Fisher Scientific, A10044) was added (10 µM) for 2 h. Cells were fixed (3.7% formaldehyde in PBS) and permeabilized (0.1% Triton X-100 in PBS). Subsequently, Click-iT reaction mix was added (1 × Click-iT cell reaction buffer, cell buffer additive, CuSO4 (Thermo Fisher Scientific, C10269), and Alexa-fluor 594 azide (2.5 µM) (Thermo Fisher Scientific, A10270)). Nuclei were stained with hoechst (15 µg/ml, Sigma). Samples were analyzed using a high-throughput, fluorescent reporter system, coupled to automated microscopy (BD Pathway 855 High Content Bioimager). Data was processed with Attovision and BD Diva software.

### Proliferation

Proliferation of BMDMs was measured on an ACEA xCELLigence (Roche). Unstimulated BMDMs (8 × 10^4^ cells) were seeded on a gold electrode implemented in a 96 wells plate and allowed to grow for 72 h. Impedance was measured hourly and used to quantify proliferation (slope of impedance increment over time) using RCTA software (version 1.2, Roche).

### Seahorse mitochondrial stress test

Cells were plated in optimal seeding density (40.000 cells/well) in an XFe96 cell culture microplate (Agilent, 102416-100) and allowed to attach for 24 h under standard culture conditions. The mitochondrial stress test was done as described previously^47^. Oxygen consumption rate was measured with a XF-96 Flux Analyzer according to manufacturer’s instruction. Oxidative phosphorylation characteristics (basal respiration, maximal respiration, ATP production, non-mitochondrial oxygen consumption) were calculated from the oxygen consumption rate differences in response to oligomycin (1 μM), FCCP (2,5 μM), and antimycin A+ Rotenone (1 μM each). After measurements, protein concentration of each well was measured using BCA kit (Pierce, Cat. No. 23227) to correct for different cell density and protein content.

### Lactate and pH measurement

Lactate and pH were determined in supernatant directly derived from BMDMs cultured under normoxic or hypoxic conditions (1% O_2_) for 72 h in standard RPMI culture medium. Measurements were performed on the GEM 4000 (Werfen, Barcelona, Spain).

### ELISA

Detection of sCA IX in plasma samples was done by commercially available Human Carbonic Anhydrase IX DuoSet ELISA (R&D Systems, MN, USA) suited to detect 31-2000 pg/ml CAIX. Briefly, detection of sCA IX in plasma was performed by sandwich ELISA using capture monoclonal antibody (200 ng/ml; 100 µl per well) coated on 96-plate and incubated overnight at room temperature. After washing and blocking (1% BSA in PBS) 100 µl of plasma sample (diluted 1:1 in reagent diluent) or standards were added and incubated for 2 h at RT. After washing, 100 µl of detection antibody (200 ng/ml) were added and incubated for 2 h at RT; streptavidin-HRP (100 µl) was used as a detector. Reaction was stopped by adding 50 µl of the stop solution and the optical density of each well was determined using a microplate reader set to 492 nm^25^.

### Genotyping

CAIX WT and KO stomach samples were used for DNA isolation and genomic DNA was amplified using REDTaq (Sigma, D8312). Primers used for CAIX PCR were the following: CAIX WT1: 5′-CCA GTC AGC TGC ATG GCC-3′, WT2: 5′-AGG AGC CTC GGG AGT CGA-3′, KO: 5′-AGG AGC AAA GCT GCT ATT GG-3′. After PCR, samples were run on a temperature gradient gel (56–65 °C) for further analysis. CAIX WT product is visualized at ~ 318 bp, while CAIX KO product is visualized ~ 400 bp.

### Real time quantitative PCR

Cells were cultured accordingly, and RNA was isolated and produced as described^48^. qPCR analyses were performed from 10 ng cDNA using SYBR green (Biorad) and gene specific primers can be found in Table 3. Two housekeeping genes (18S and cyclophilin) were used to correct for different mRNA quantities between samples.

**Table 3:** Primers for RT-PCR.

Primer	FW	RV
18S	GTAACCCGTTGAACCCCATT	CCATCCAATCGGTAGTAGCG
Cyclophilin	CAAATGCTGGACCAAACACAA	TTCACCTTCCCAAAGACCACAT
iNOS	CCTGGTACGGGCATTGCT	GCTCATGCGGCCTCCTTT
IL6	CTGCAAGAGACTTCCATCCAGTT	GAAGTAGGGAAGGCCGTGG
TNF	CATCTTCTCAAAATTCGAGTGACAA	TGGGAGTAGACAAGGTACAACCC
A20	CAAGGGCTTTTGCACTCTATGTT	GGCACGGGACATTGTTCTG
IL10	TTTGAATTCCCTGGGTGAGAA	CTCCACTGCCTTGCTCTTATTTTC
CD206	TGCAAAGGACTGAAAGGAAACC	CCAGTCCAGGCATTGAAAGTG

### Human tissue collection

Multiple human plaque or plasma collections were used: Maastricht Pathology Tissue Collection (MPTC) including the Maastricht human plaque study (MAASHPS^49^, and Maastricht human plasma cohort^28^, the Athero-Express biobank study (www.atheroexpress.nl)^31^, and plasma samples of Cohort On Diabetes and Atherosclerosis Maastricht (CODAM)^50–52^, for analysis of plaque protein and mRNA levels using immunohistochemistry, microarrays, and western blot analysis and plasma CAIX levels.

All patient material collection, storage, and use of tissue and patient data were performed in agreement with the Dutch Code for Proper Secondary Use of Human Tissue (http://www.fmwv.nl). The studies all comply with the Declaration of Helsinki, and the local Medical Ethics Committee in accordance with national regulations approved use of this tissue. To be more precise, MPTC, Maastricht human plaque study, Maastricht human plasma cohort and CODAM were approved by the Medical Ethics Committee of the Maastricht University Medical Centre. The Athero-Express study was approved by Medical Ethics Committee of University Medical Center Utrecht. All included patients gave their written informed consent.

*Maastricht human plaque study (MAASHPS) cohort* Human atherosclerotic plaque samples or serum were obtained from carotid artery lesions from patients undergoing endarterectomy (Department of Vascular Surgery, Maastricht University Medical Center and Zuyderland Medical Center, Sittard-Geleen, the Netherlands). Seven patients were injected with pimonidazole prior to surgery to detect tissue hypoxia^1^. The MaasHPS (Maastricht Human Plaque Study) consists of 22 patients, who underwent carotid endarterectomy, and comprised the intra-patient, paired comparison of stable segments with thick fibrous cap atheroma’s to unstable segments containing intraplaque hemorrhage in the same plaques. Plaques were sequentially divided in alternating samples used for formalin fixation, or snapfrozen for protein or transcriptomic analysis by microarray. Microarray analysis was used to study whole plaque mRNA expression as described elsewhere^53^. Classification of plaque stage was done according to Virmani et al.^54^ by two investigators independently. After RNA quality check, and re-classification after quantitative morphometry, 16 stable, and 27 unstable segments were included in the analysis. Pearson correlations were calculated between CAIX mRNA expression with several plaque traits in unstable plaques, as determined by plaque histology and immunohistochemistry. For all immunohistochemical purposes, two slides per patient for each stable and unstable segment were used, hence four slides in total. Adjacent tissue sections were phenotyped extensively for plaque size, necrosis, inflammation (CD68, CD3, arginase, iNOS), SMCs and fibroblasts (αSMA), collagen (Sirius red), macro- and micro-calcification (Alizarin red), and angiogenesis (CD31+ microvessel density, newly formed CD105+ Cd31+ double-positive microvessels, αSMA + CD31 + double-positve mature microvessels, Lyve + lymphatic density). Analysis of each individual staining was performed using Leica Qwin software, and values averaged per segment, resulting in two values per patient, one for the stable and one for the unstable segment.

*Maastricht human plasma cohort* A description of the participants and eligibility criteria of enrolled patients in the original study were described elsewhere^28^. In short, we selected plasma of a total of 63 patients with either stable or unstable carotid artery plaques, as determined by histology^54^.

*Athero-express biobank study* In the Athero-Express Biobank Study, carotid plaques were collected from symptomatic patients for histology and protein lysis^31^. Tris lysates of 64 human plaques were used for CAIX western blot analysis of which 48 (75%) men, 69 ± 8 years old, 27 (42%) participants with a secondary event, 42 (66%) plaques with IPH, 26 (40.6%) atheroma’s, 33 (51.6%) fibrous plaques and 5 (7.8%) fibroatheromas. Patient and plaque characteristics in participants with and without plaque-resident CAIX are described in supplemental table S3.

*CODAM* The CODAM cohort was designed to study associations between diabetes, atherosclerosis and other cardiovascular diseases. An extensive description of the cohort and execution and description of used measurements can be found elsewhere^50–52^. In short, 572 participants with, or at risk for, diabetes mellitus type 2 were enrolled in this study, and followed for 7 years. Any cardiovascular outcomes were documented, and blood was withdrawn at enrollment and after 7 years follow-up. In CODAM, CVD was defined as the occurrence of at least one of the following: previous myocardial infarction (MI); coronary bypass; percutaneous coronary intervention (PCI); stroke or transient ischemic attack reported by questionnaires; signs on a 12-lead electrocardiogram of MI or ischemia, traumatic limb amputation; and/or an ankle brachial index < 0.9. Cardiovascular events (CVE), comprised of MI, stroke, coronary bypass and/or PCI, as reported^55^. An overview of all human cohorts and corresponding information can be found in Table 4.

**Table 4:** Overview human patient cohorts.

Cohort	Number of patients	Collected material	Assays performed	Figure/Table
MaasHPS	22 (16 stable vs. 27 ustable plaque segments)	Snapfrozen material, tissue sections	Microarray, immunohistochemistry, Pimonidazole hypoxia	Figure 1
Maastricht human plasma cohort	63	Plasma	ELISA	Figure 4
AtheroExpress	64	Plaque protein lysates	Western blot	Figure 4
CODAM	572	Plasma	ELISA	Tables 1, 2, Supplemental tables S2-3

### Human carotid protein isolation and western blot

Human carotid plaques were snap-frozen immediately after collection. Sample preparation procedures were carried out on dry ice. Carotid plaques were divided in smaller pieces (± 0.5 cm) and manually grinded under constant addition of liquid nitrogen. The resulting tissue dust was incubated with 500 µl TRIS lysis buffer and EDTA-free protease inhibitor cocktail (Roche, 04693159001). Subsequently, protein liberation was further enhanced by crushing grinded tissue in a Beadbeater, and sonicating the sample for 2 min. Upon centrifugation (maximal speed, 5 min) supernatant was collected and protein concentration was determined using BCA kit (Pierce, Cat. No. 23227). Pre-cast gels (ExpressPlus PAGE gel 8–16%, genscript, M81612) were used for protein seperation, and transferred to a nitrocellulose membrane. CAIX was detected using primary antibody M75 (1:3000) followed by HRP-labeled secondary antibody incubation (Jackson, 715-035-150). Signal was developed using SuperSignal West Femto Maximum Sensitivity Substrate (Thermo Fisher scientific, 34095) and visualized using a digital scanner. Signal intensity was quantified using ImageJ Gel Analyzer software and normalized for total protein content using intensity of Ponceau S. Each gel contained the same plaque sample, allowing normalization between gels.

### Immunohistochemical staining

Human carotid sections (4 µm) were deparaffinized, stained with CD68 mouse—anti-human (Dako, GA60961-2) or iNOS rabbit—anti-human (Abcam, ab3523) antibody and stained with vector red alkaline phosphatase kit (Vector, SK-5100). Subsequently, antigen retrieval was performed using citrate buffer pH6.0 (Dako REAL target retrieval, Dako). CAIX was stained with M75 primary antibody and visualized using vector blue alkaline phosphatase kit (Vector, SK-5300). Hypoxia was detected, in patients who received a pimonidazole injection prior to endarterectomy, using HRP labeled rabbit—anti-pimonidazole antibody (PAb2627, NPI). For iNOS detection, rabbit-anti human iNOS antibody (ab3523, Abcam) was used. Hypoxia and iNOS staining were visualized using AEC + Substrate-Chromogen (K3461, Agilent). Prior to Entallan mount, slides were dehydrated using Imsol (diluted 1:5) on the hot plate (37 °C).

### Statistical analysis

sCAIX plasma values were, even after log transformation, not normally distributed; moreover sCAIX was only detectable in 80 of 572 plasma samples. Therefore, we performed subsequent regression analyses with sCAIX as a dichotomous independent variable (sCAIX detectable yes/no). Cross-sectional analysis was performed to assess prevalent CVD and CVE, as defined in CODAM. Logistic regression (IBM SPSS Statistics, Version 25.0.) was used to test association between sCAIX and presence of CVD or CVE. To this end, the following models were employed. Model 1: Crude, no adjustments. Model 2: model 1 + adjustments for sex and age. Model 3: model 2 + adjustments for smoking (status [current, former, never] & packyears), medication (lipid-modifying y/n, anti-HT y/n, glucose-lowering y/n), glucose metabolism status (IGM y/n, DM2 y/n). Linear regression was performed to study the association between sCAIX (detectable yes/no) and Intima-media thickness of the carotid artery (cIMT) and ankle-arm index (AAIx). To assess prognostic value of sCAIX, we performed a prospective analysis, with outcome measures CVD and CVE as determined on the end of follow-up period. Here, similar logistic regression models were employed. Only participants that did not have CVD (n = 317) or CVE (n = 369) at time of inclusion were included in this analysis.

Distribution of plaque-resident CAIX was also not normal, and positively skewed. Square root transformation rescued skewness, but CAIX distribution remained not normal as shown by Shapiro–Wilk normality test. Non-parametric Mann–Whitney rank sum tests were done to test if CAIX was statistically different between plaques with a single or secondary event, with and without IPH, and between atheroma’s and fibrous plaques. In addition, dichotomous analysis of plaque CAIX using Fisher’s exact test was performed.

All in vitro data are presented as mean + SEM, with *p-value < 0.05, **p-value < 0.01, ***p-value < 0.0001. All parameters were analyzed using independent sample tests and were tested for normal distribution using Shapiro–Wilk normality test. Parameters with two groups were compared with student’s t-test or Mann–Whitney rank-sum test.

## Supplementary Information

Supplementary Information.
