# Supplementary material for: The hypoxia-sensor carbonic anhydrase IX affects macrophage metabolism, but is not a suitable biomarker for human cardiovascular disease
Source: Sci Rep. 2021 Jan 11;11:425. doi: 10.1038/s41598-020-79978-5 (PMC7801702; doi:10.1038/s41598-020-79978-5)

Online supplement to :

"The hypoxia-sensor carbonic anhydrase IX affects macrophage metabolism, but is not a suitable biomarker for human cardiovascular disease"

Demandt JAF,[1,2] Dubois LJ,[3] van Kuijk K, [1,2] Zaťovičová M,[4] Jin H,[1,2] Parkkila S,[5,6] van der Laan SW,[7] Jelenska L,[4] Mees BME,[1,8] Reutelingsperger CPM,[1,9] Cleutjens KBJM,[1,2] van der Kallen CJH,[1,10] Schalkwijk CG,[1,10] van Greevenbroek MMJ,[1,10] Biessen EAL,[1,11] Pasterkamp G,[7] Pastoreková S,[4] Stehouwer CDA,[1,9] Sluimer JC[1,2,12]

Supplemental Table S1. Correlation *CAIX* mRNA to apoptosis pathway (MSigDB_HALLMARK_APOPTOSIS) in unstable plaque segments

Gene	Correlation	p.value
ENO2	0.58	0.002
IL1B	0.57	0.002
CDK2	0.42	0.027
GADD45B	0.43	0.024
GNA15	0.43	0.025
PEA15	0.44	0.023
PPP3R1	0.45	0.017
PTK2	-0.42	0.031
TXNIP	0.42	0.031
CD14	0.40	0.041
BCL2L1	0.28	0.159
BTG3	0.36	0.067
CAV1	-0.31	0.117
CDKN1A	0.31	0.113
CTH	-0.31	0.113
DFFA	-0.32	0.108
DNAJA1	-0.36	0.063
EREG	-0.30	0.122
GSTM1	-0.29	0.149
HMGB2	0.31	0.112
IL1A	0.34	0.081
NEDD9	0.31	0.120
NEFH	0.29	0.149
PDGFRB	-0.31	0.115
PLCB2	0.31	0.111
RELA	0.28	0.156
ROCK1	-0.34	0.080
SLC20A1	0.30	0.134
SOD1	-0.37	0.056
TGFBR3	-0.31	0.112
TIMP1	-0.28	0.159
TIMP2	-0.29	0.135
VDAC2	-0.30	0.135
BNIP3L	0.27	0.172
CD44	0.27	0.178
PLAT	0.27	0.175
BMP2	-0.26	0.193
IFNGR1	0.26	0.193
PAK1	0.24	0.228
RHOT2	0.24	0.234
AIFM3	0.15	0.445
ANKH	-0.20	0.327
ANXA1	-0.21	0.291
APP	0.10	0.625

ATF3	-0.20	0.306
AVPR1A	0.09	0.641
BAX	0.10	0.612
BCL10	-0.12	0.546
BCL2L10	-0.18	0.371
BCL2L11	-0.09	0.662
BCL2L2	0.09	0.656
BGN	0.06	0.753
BIK	0.06	0.768
BIRC3	0.14	0.486
BTG2	-0.15	0.462
CASP1	0.07	0.742
CASP3	0.09	0.671
CASP4	0.08	0.700
CASP6	-0.16	0.436
CASP7	-0.08	0.679
CASP8	-0.17	0.392
CASP9	0.07	0.714
CCND2	0.22	0.269
CD2	0.07	0.734
CD38	-0.15	0.444
CD69	0.11	0.571
CDC25B	0.14	0.499
CDKN1B	-0.10	0.613
CREBBP	-0.16	0.439
DAP	-0.08	0.700
DAP3	-0.06	0.750
DCN	0.08	0.692
DDIT3	-0.06	0.763
DNAJC3	-0.15	0.451
DPYD	-0.09	0.649
EGR3	-0.07	0.718
ERBB2	-0.11	0.590
ERBB3	0.09	0.640
F2	-0.14	0.491
FAS	-0.11	0.599
FASLG	0.09	0.671
FDXR	0.15	0.468
FEZ1	-0.17	0.397
GADD45A	-0.08	0.686
GCH1	0.08	0.680
GPX1	0.14	0.491
GPX3	0.19	0.340
GPX4	-0.14	0.493
GSN	0.09	0.672
GSR	0.09	0.668
GUCY2D	-0.22	0.262
HGF	-0.19	0.345

HMOX1	-0.14	0.477
HSPB1	-0.21	0.299
IFNB1	0.13	0.515
IGF2R	-0.09	0.671
IGFBP6	-0.08	0.693
IL6	0.15	0.440
IRF1	-0.10	0.604
JUN	-0.08	0.702
LEF1	-0.08	0.685
LGALS3	-0.17	0.385
LMNA	-0.16	0.414
LUM	0.19	0.331
MCL1	0.08	0.700
PPP2R5B	0.20	0.324
PPT1	0.10	0.607
PRF1	0.13	0.534
PSEN2	-0.21	0.285
RARA	0.07	0.741
RETSAT	0.12	0.537
RHOB	0.08	0.684
RNASEL	0.10	0.622
SMAD7	-0.19	0.338
SOD2	0.06	0.767
SQSTM1	-0.14	0.498
TAP1	0.06	0.759
TGFB2	-0.16	0.414
TIMP3	-0.14	0.493
TNF	0.19	0.347
TOP2A	0.09	0.650
TSPO	-0.12	0.567
WEE1	-0.19	0.332
CLU	-0.05	0.794
H1F0	0.05	0.796
PSEN1	0.05	0.793
ADD1	0.01	0.972
BCAP31	-0.04	0.851
BID	0.00	0.989
BMF	0.04	0.860
BRCA1	-8.10	1.000
CASP2	-0.02	0.938
CCNA1	0.00	0.998
CCND1	-0.03	0.891
CFLAR	-0.01	0.961
CTNNB1	-0.01	0.941
CYLD	-0.02	0.936
DIABLO	-0.01	0.968
DNM1L	0.00	0.992
EBP	-0.04	0.844

EMP1	0.03	0.896
ETF1	-0.02	0.915
F2R	0.03	0.888
IER3	0.00	0.994
IFITM3	0.00	0.984
IL18	0.01	0.941
ISG20	0.04	0.832
KRT18	0.05	0.818
MADD	-0.05	0.818
MGMT	0.02	0.941
MMP2	-0.01	0.956
PDCD4	0.00	0.996
PMAIP1	0.01	0.957
SAT1	0.01	0.966
SATB1	0.04	0.838
SPTAN1	0.04	0.856
TNFRSF12A	0.00	0.986
TNFSF10	0.03	0.874

Supplemental Table S2. Patient demographics and exploratory data of CODAM cohort

General/metabolic measurements	sCAIX detectable N=80	sCAIX undetectable N=492	P-value
Age (years)	60.9 ± 6.9	59.4 ± 6.9	0.085
Sex, N Men (%)	47 (58.8 %)	304 (61.8%)	0.605
BMI (kg/m2)	28.6 ± 4.7	28.6 ± 4.2	0.872
Waist (cm)	99.2 ± 12.0	99.3 ± 11.9	0.948
SBP (mmHg)	141.4 ± 19.5	140.2 ± 19.3	0.618
DBP (mmHg)	81.8 ± 9.5	81.9 ± 9.3	0.942
Fasting Plasma Glucose (mmol/l)	6.3 ± 1.8	6.0 ± 1.4	0.214
Total cholesterol (mmol/l)	5.1 ± 1.0	5.2 ± 1.0	0.407
HDL-C (mmol/l)	1.21 ± 0.35	1.18 ± 0.35	0.650
TG (mmol/l)	1.4 [0.9; 1.8]	1.4 [1.0; 2.0]	0.085
Current smoker, Y (%)	13 (16.2 %)	101 (21.1%)	0.320
Packyears*	5.3 [0; 30.6]	15.0 [0; 31.1]	0.053
Smoking, Current/Former/Never (%)	13 / 32 / 35 (16.2/40.0/43.8)	101 / 251 / 127 (21.1/2.4/26.5)	0.007
NGM/IGM/DM2 (%)	47 / 10 / 23 (58.8/12.5/28.7)	252/117/123 (51.2/23.8/25.0)	0.079
DM2, Y (%)	23 (28.7 %)	123 (25.0 %)	0.476
Glucose medication, Y (%)	18 (22.5 %)	58 (11.8 %)	0.009
Lipid medication, Y (%)	11 (10.2 %)	97 (19.7 %)	0.206
BP medication, Y (%)	23 (28.7 %)	197 (40.0%)	0.054
Rheumatoid arthritis, Y (%)	7 (9.1 %)	43 (8.8 %)	0.944
Cancer, Y (%)	3 (15.0 %)	17 (3.5 %)	0.905
**Cardiovascular measurements**			
CVD, Y (%)	20 (25.0 %)	138 (28.1 %)	0.565
CVE, Y (%)	11 (13.8 %)	78 (15.9 %)	0.625
IMT**	0.80 ± 0.19	0.77 ± 0.15	0.140
AAIx***	1.09 ± 0.13	1.10 ± 0.13	0.548
PAD (=AAix<0.9) , Y (%)	5 (6.2 %)	23 (4.7 %)	0.548
Plaque present, Y (%) (available for only 366)	3 (6.7 %)	14 (4.4 %)	0.491
**Other measurements**			
MMP2 (ng/ml)	157 [141; 177]	146 [129; 163]	<0.001
MMP9 (ng/ml)	14.3 [10.7; 20.1]	14.5 [11.4; 20.1]	0.448
IL6 (ng/l)	1.6 [1.1; 2.5]	1.6 [1.2; 2.2]	0.959
eGFR (ml 1.73 m2 / min)	90.7 [80.3; 100.2]	90.5 [78.1;101.3]	0.775

* Values for packyears were analyzed in 560 participants. **Values for cIMT were analyzed in 504 participants. ***Values for AAIx were analyzed in 541 participants. Data are presented as mean ± SD (normally distributed variables), median [interquartile range] (skewed variables) or proportion (%, categorical variables). BMI; body mass index. SBP; systolic blood pressure. DBP; diastolic blood pressure. HDL-C; high-density lipoprotein cholesterol. TG; triglycerides. NGM; normal glucose metabolism. IGM; impaired glucose metabolism. DM2; diabetes mellitus type 2. CVD; cardiovascular disease. CVE; cardiovascular event. IMT; intima-media thickness. AAIx; ankle-arm index. PAD; peripheral artery disease. MMP2/9; matrix metalloproteinase 2/9. IL6; interleukin 6. eGFR; estimated glomerular filtration rate. Y, counts for yes. p-Values were obtained by ANOVA, ANOVA of ln-transformed variables or Pearson Chi-square.

Supplemental Table S3. Association of sCAIX with incident CVD and CVE

CVD	OR	95%; CI	p-value
Model 1	0.90	0.410; 1.965	0.787
Model 2	0.87	0.393; 1.904	0.719
Model 3	0.82	0.359; 1.878	0.640
**CVE**	**OR**	**95%; CI**	**p-value**
Model 1	0.62	0.233; 1.629	0.329
Model 2	0.59	0.223; 1.580	0.296
Model 3	0.63	0.229; 1.737	0.373

Logistic regression analysis using 317 and 369 subjects that were free of CVD or CVE at time of inclusion, respectively. sCAIX was treated as dichotomous independent variable (detectable yes/no). β indicates mutation of dependent variable if CAIX is detectable (yes). Model 1: Crude, no adjustments. Model 2: model 1 + adjustment for sex and age. Model 3: model 2 + adjustments for smoking (status [current, former, never] & packyears), medication (lipid-modifying y/n, anti-HT y/n, glucose-lowering y/n), glucose metabolism status (IGM y/n, DM2 y/n).

Supplemental Table S4 Athero-Express patient demographics and plaque traits in subjects with and without detectable plaque-resident CAIX

Patient baseline demographics		CAIX detectable N=34	CAIX undetectable N=30
Age (years)		68.1±8.6	70.7±7.3
Sex, Men N (%)		27 (79.4 %)	21 (70.0 %)
Primary symptoms N (%)	Stroke	8 (23.5 %)	8 (26.7 %)
	TIA	7 (20.6%)	6 (20.0 %)
	Other	19 (55.9%)	16 (53.3 %)
Total cholesterol (mmol/l)		4.4±1.1	5.0±1.3
LDL-C (mmol/l)		2.5±0.9	3.2±1.1
HDL-C (mmol/l)		1.2±0.3	1.4±0.3
TG (mmol/l)		1.6±0.8	1.2±0.5
BMI (kg/m2)		154.1±30.5	172.0±31.1
SBP (mmHg)		78.8±12.9	85.0±17.5*
DBP (mmHg)		26.5±4.1	26.0±3.0
Smoking (packyears)		31.1±23.2	24.3±18.1
DM2, Y (%)		6 (17.6 %)	3 (10.0%)
Hypertension , Y (%)		29 (85.3%)	26 (86.7%)
BP medication, Y (%)		27 (85.3 %)	24 (80.0 %)
Antiplatelet medication, Y (%)		33(97.1%)	27 (90.0%)
Lipid medication, Y (%)		29 (85.3%)	22 (73.3%)
**Patient follow-up**			
Second event, Y (%)		16 (47.1%)	11 (36.7%)
**Plaque phenotype**			
IPH, Y (%)		24 (70.6%)	18 (60.0%)
Macrophage N (%)	Heavy/moderate	11 (32.4%)	14 (46.7%)
Collagen N (%)	Heavy/moderate	29 (85.3%)	22 (73.3%)
Plaque type N (%)	Atheroma	12 (35.3%)	14 (46.7%)
	Fibroatheroma	1 (2.9%)	4 (13.3%)
	Fibrous	21 (61.7%)	12 (40.0%)

Data are presented as mean ± SD or proportion (%, categorical variables). BMI; body mass index. SBP; systolic blood pressure. DBP; diastolic blood pressure. LDL-C; low-density lipoprotein cholesterol, HDL-C; high-density lipoprotein cholesterol. TG; triglycerides. DM2; diabetes mellitus type 2. Collagen/macrophage categories Heavy-moderate vs no-minor. Data were analyzed using Mann Whitney rank sum or Fisher's Exact test, and corrected for multiple testing. Y, counts for category "yes", N counts. * P-value < 0.05, all others not significant.

SUPPLEMENTAL FIGURE LEGENDS

Supplemental Figure S1

Genotyping of CAIX DNA in CAIX WT and KO stomach tissue. Expected band WT ~318bp, KO ~400bp.

Supplemental Figure S2

Representative immunohistochemical picture of CAIX protein expression in unstable plaque segments of the MaasHPS cohort (N=27). CAIX in blue, nuclei in red. Depicted as 50x magnification (A) and 200x magnification (B) of the red squared area.

Supplemental Figure S3

Full western blots and PonceauS for figure 4B

Supplemental Figure S4

Full western blots and PonceauS for figure 4D

Supplemental Figure S1

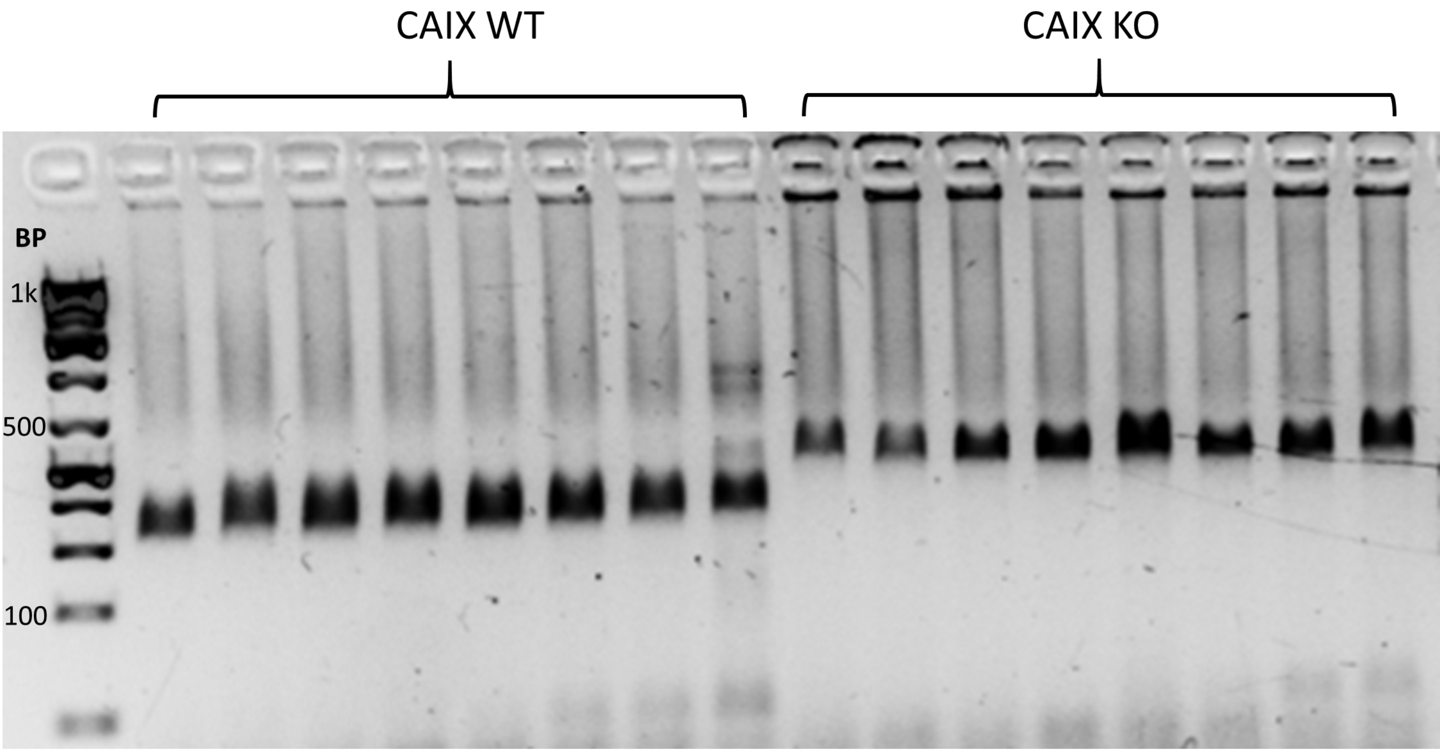

Supplemental Figure S2

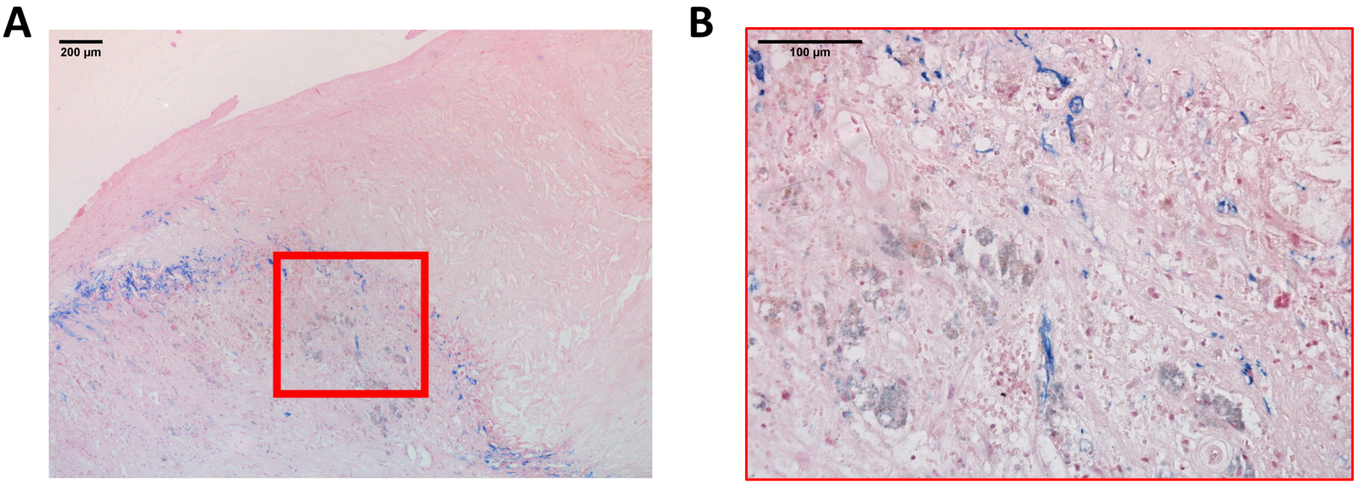

Supplemental Figure S3

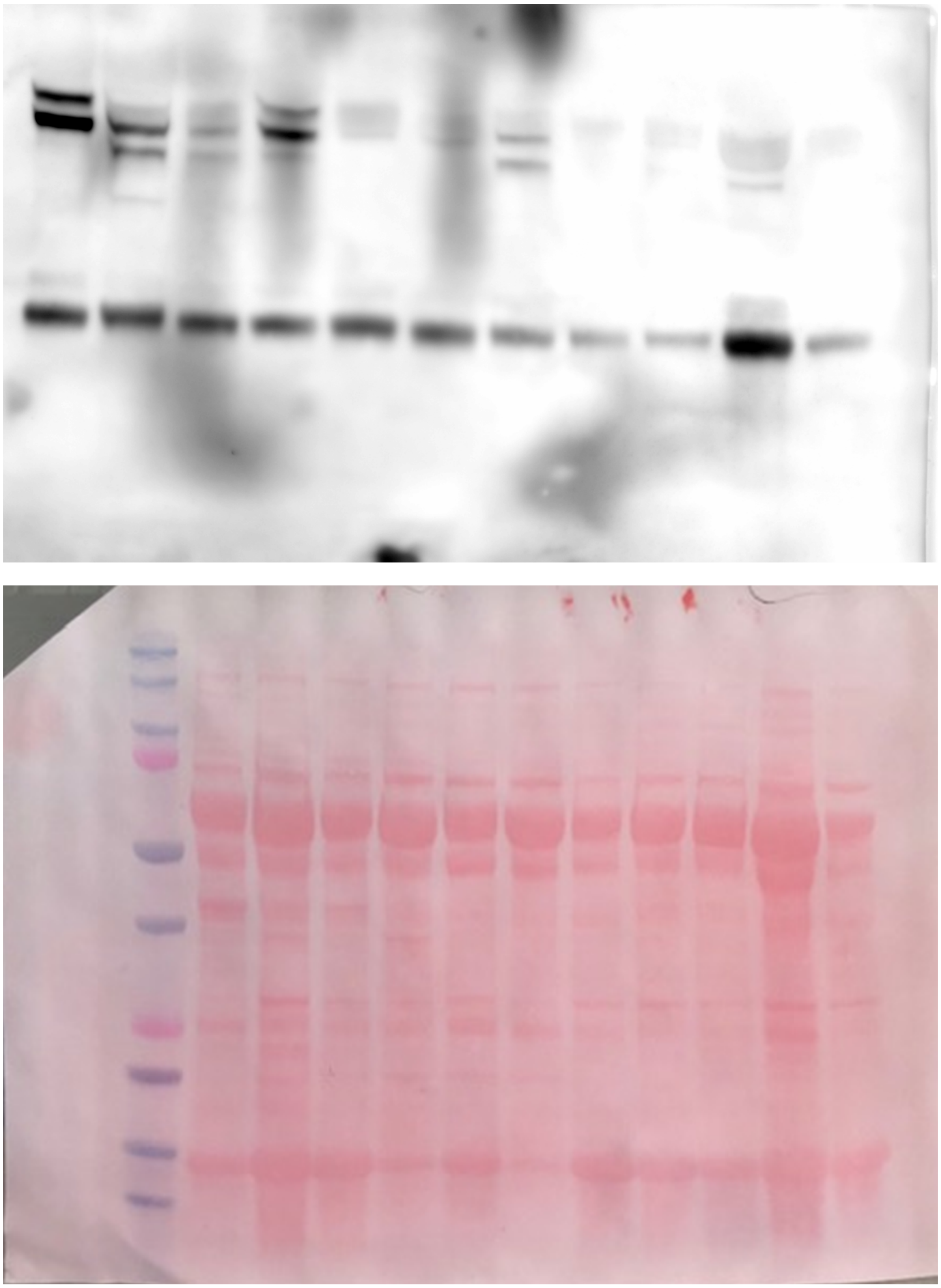

Supplemental Figure S4

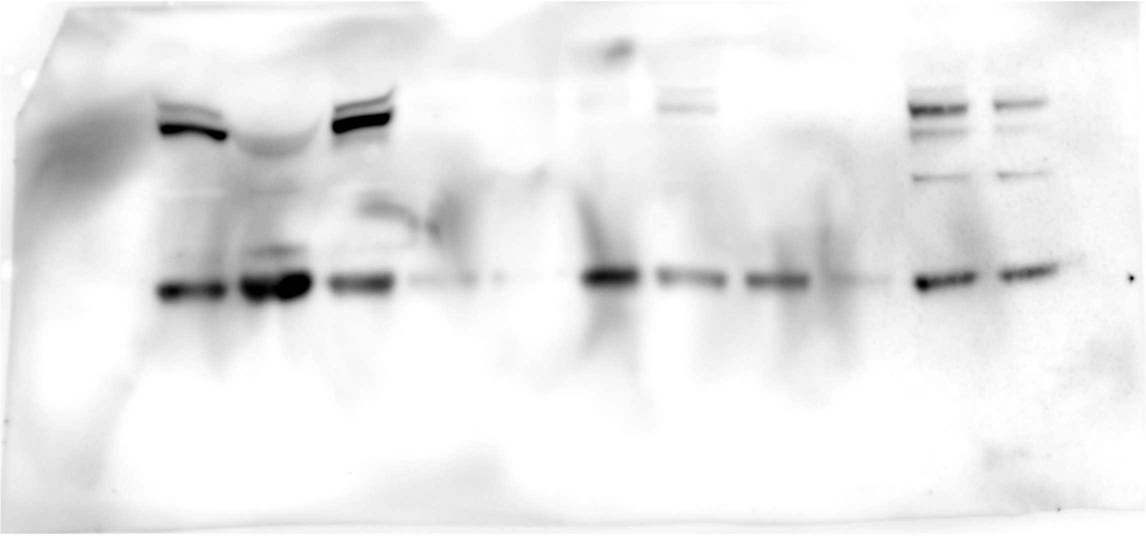

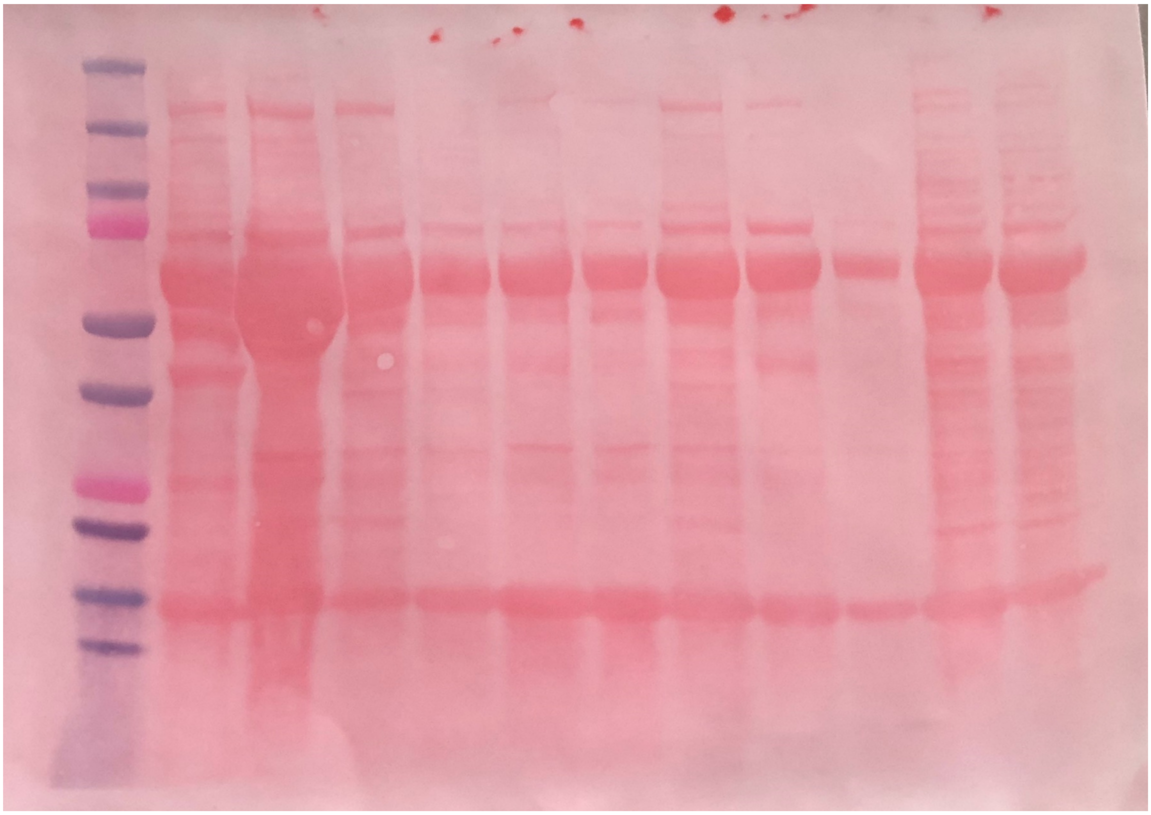

Supplement: Supplementary file 1 — Supplementary Information. [file 41598_2020_79978_MOESM1_ESM.pdf]
